# Over-night monitoring in intensive care unit and short-term monitoring in post anesthesia care unit costs analysis after elective hepato-pancreatic–biliary surgery: a retrospective study

**DOI:** 10.1007/s13304-025-02405-9

**Published:** 2025-12-02

**Authors:** Paolo Vincenzi, Federico Mocchegiani, Andrea Benedetti Cacciaguerra, Diletta Gaudenzi, Daniele Nicolini, Paolo Cerchiara, Federica Bartolini, Nadia Moroni, Elisabetta Cerutti, Marco Vivarelli

**Affiliations:** 1 Division of HPB and Abdominal Transplant Surgery, Department of Gastroenterology and Transplants, Azienda Ospedaliero-Universitaria Delle Marche, 60126 Ancona, Italy; 2 Division of HPB and Abdominal Transplant Surgery, Department of Experimental and Clinical Medicine, Polytechnic University of Marche, 60126 Ancona, Italy; 3 Division of Anesthesia, Transplant and Surgical Intensive Care Unit, Department of Emergency, Azienda Ospedaliero-Universitaria Delle Marche, 60126 Ancona, Italy

**Keywords:** Post anesthesia care unit, Intensive care unit, Hepato-pancreatic–biliary surgery, Costs, Postoperative monitoring

## Abstract

The post anesthesia care unit (PACU) was introduced to optimize intensive care unit (ICU) bed availability for patients requiring specialized perioperative care, providing comprehensive monitoring during the recovery phase in high-risk surgical patients. However, though several advantages of PACUs have been clearly demonstrated, no previous studies have investigated the overall costs. This retrospective study aimed at comparing the costs of postoperative monitoring in PACU and ICU after elective hepato-pancreatic–biliary (HPB) surgery at our hospital. A retrospective cohort study was conducted between January 2022 and December 2024. The investigations periods were divided according to the institution of PACU at our institution: before (January 1, 2022–May 31, 2023) and after (June 1, 2023–December 31, 2024). Three groups were identified: ICU 1 and 2, including all pt admitted to ICU before and after the institution of PACU, respectively, and PACU, including all pt admitted to PACU. The primary outcome was the cost of monitoring/pt. Secondary outcome was the overall hospital length of stay (LOS). 171 consecutive pt undergoing HPB surgery were included and divided into 49 pt belonging to ICU 1 group, 60 to ICU 2 and 62 to PACU. Multivariable analysis of monitoring cost revealed the following independent predictors: postoperative surveillance in PACU (*P* < 0.0001), duration of monitoring (*P* < 0.0001), male gender (*P* = 0.028) and severity of complications (*P* = 0.001), the latter resulting the only significant risk factor for prolonged LOS (*P* < 0.0001). PACU has emerged as a safe and efficient alternative to ICU in the postoperative surveillance of HPB surgical patients, with associated significant cost savings. Further research is required to identify specific criteria addressing the individual needs for postoperative admission to ICU or PACU.

**Supplementary Information:**

The online version contains supplementary material available at 10.1007/s13304-025-02405-9.

## Introduction

The post anesthesia care unit (PACU), also known as enhanced perioperative care unit (EPCU), is an essential component of modern surgical care, aiming at providing a safe and comfortable environment for patients as they recover from anesthesia after surgery [1].

This facility, typically located near the operating room (OR) and staffed by highly trained nurses and anesthesiologists, is specifically dedicated to provide specialized perioperative care to high-risk surgical patients, ensuring advanced monitoring, pain management, and early recognition of immediate post-operative complications, leading eventually to improved surgical outcomes and reduced overall length of stay (LOS) [2, 3].

PACUs have received increasing attention over recent years as an important measure to decrease the pressure on intensive care units (ICUs), improving resource allocation by addressing ICU’s limited capacities to critically ill patients. In addition, the duration of stay in the PACU is typically tailored to the individual patients’ needs, targeting faster recovery after surgery, contrarily to most critical care units (CCUs), where patients routinely are monitored at least one night after surgery, even in absence of early complications [4].

Accordingly, PACUs have become a highly effective and cost-efficient alternative to the ICU, reducing the financial burden on healthcare systems [1].

However, to the best of our knowledge, no studies so far have investigated the potential benefits of the PACU in terms of cost savings when compared to the ICU in a population of patients undergoing elective hepato-pancreato–biliary (HPB) surgery. Thus, in the current study, we hypothesized that postoperative care in the PACU is associated with a reduction in costs, without increasing the postoperative morbidity rate and the overall LOS.

To answer this question, we realized a model of cost calculation, both for the PACU and the ICU, assisted by cost accounting and management controllers.

## Materials and methods

### Study design and endpoints

This study was designed as a retrospective cohort study comparing three separate groups of consecutive patients undergoing elective HBP surgery in a tertiary teaching regional hospital before the institution of the PACU (January 1, 2022–May 31, 2023) and after the institution of the PACU (June 1, 2023–December 31, 2024).

The inclusion criteria were as follows:

1. Age ≥ 18 years;
2. Patients undergoing elective HPB surgery for the following diseases: primary benign and malignant liver tumors, liver metastases, benign liver diseases (hepatic echinococcosis, Caroli disease), primary pancreatic tumors (pancreatic adenocarcinoma, intraductal papillary mucinous neoplasm, mucinous cystadenoma, neuroendocrine tumor), duodenal adenocarcinoma;
3. The different surgical interventions performed for the above-mentioned conditions were identified through the procedural codes according to the International Classification of Diseases, 9th Revision, Clinical Modification (ICD-9-CM) [5], as listed in Appendix A (Table A1).
4. Patients directly admitted to the PACU or the ICU after surgery.

The exclusion criteria were as follows:

1. Age < 18 years;
2. Patients undergoing urgent HPB surgery, including trauma;
3. Patients directly admitted to the surgical ward after surgery and not requiring ICU or PACU monitoring;
4. Patients transferred from PACU to ICU after surgery;
5. Patients with a LOS in ICU ≥ 24 h, due to the development of early postoperative complications.

In our hospital, the PACU has been established on June 1, 2023, and since then, organized to provide intensive hemodynamic monitoring and treatments to stabilize vital functions, including vasopressor support, after a wide range of surgeries, and under the supervision of an attending anesthesiologist. Therefore, we identified two separate groups of patients directly admitted to the ICU for postoperative monitoring after elective HBP surgery in two different periods, before (historical controls) and after (contemporary controls) the institution of the PACU.

In summary, three main groups were identified:

1. Group one, named “ICU 1”, composed of all patients directly admitted to the ICU after surgery between January 1, 2022, and May 31, 2023;
2. Group two, named “PACU”, composed of all patients directly admitted to the PACU after surgery between June 1, 2023, and December 31, 2024;
3. Group three, named “ICU 2”, composed of all patients directly admitted to the ICU after surgery between June 1, 2023, and December 31, 2024.

ICU 1 group was compared to PACU group and likewise ICU 2 group was compared to PACU group.

**Fig. 1:**
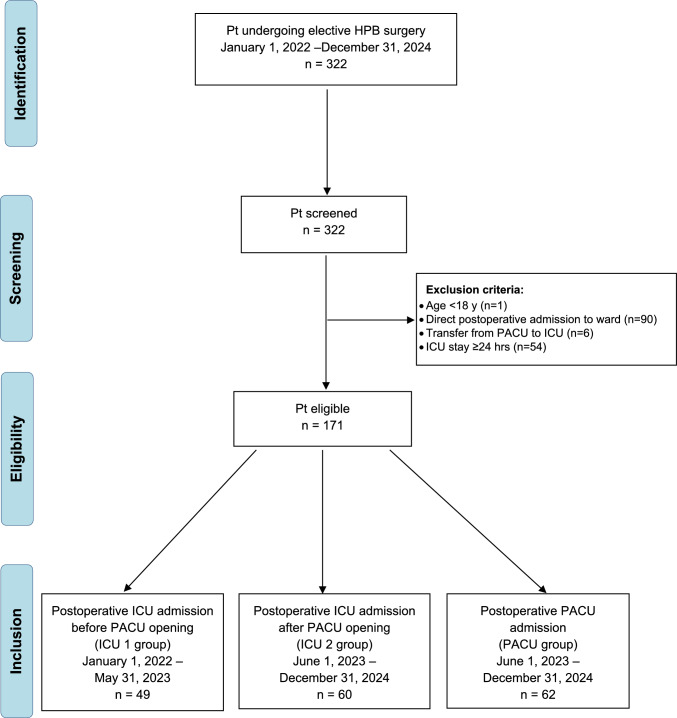
Flow diagram of this study

A flow diagram, describing the inclusion and exclusion criteria, is shown in Fig. 1.

The primary endpoint of the study was to evaluate the mean cost to patient of the postoperative monitoring in the ICU and the PACU.

The secondary endpoint was to evaluate the mean overall postoperative LOS in the three different groups.

All the baseline and outcome variables analyzed are listed in Appendix A (Table A2).

Data were obtained from a prospectively maintained electronic database and complemented by review of clinical charts.

The Strengthening the Reporting of Observational studies in Epidemiology (STROBE) guidelines were used as a checklist in ensuring high-quality presentation of the present study [6].

### Sample size calculation

The sample size was calculated to detect a significant difference of 100 € for each patient admitted to the PACU, compared to the ICU, in the costs of postoperative monitoring, between the two treatment groups. Assuming a Type I error (α) of 5% and a Type II error (β) of 20% (corresponding to 80% power), the minimum number of participants required was 68 per group.

Likewise, the sample size was calculated for the secondary outcome investigated, to identify a significant difference of 4 days in the overall postoperative LOS for each patient admitted to the PACU, compared to the ICU. Assuming a Type I error (α) of 5% and a Type II error (β) of 20% (corresponding to 80% power), the minimum number of participants required was 57 per group.

### Postoperative care setting

Selection for admission to either the ICU or the PACU was left to the discretion of the anesthesiologist at the preoperative outpatient clinic in combination with the estimation of the anesthesiologist and the perioperative team including the surgeon on the day of surgery in case of unforeseen events.

The characteristics of the ICU and the PACU in our institution are presented in Appendix A (Table A3).

The ICU has twelve beds for patients, both medical and surgical, available day and night during the entire week. Continued postoperative care is managed by specialized nurses with a nurse-to-patient ratio of 1:2 and a critical care physician is responsible for their management, with a physician-to-patient ratio of 1:4. Patients undergoing elective HPB surgery generally stay overnight and in absence of early postoperative complications, are discharged the first postoperative morning to the SW at the discretion of the supervising attending.

In contrast to the ICU, the PACU is located inside the OR department, has four beds during weekdays, only from 8 a.m. to 8 p.m. and is closed on the weekends (Saturday and Sunday) and holidays. Similarly, continued postoperative care at the PACU is managed by specialized nurses with a nurse-to-patient ratio of 1:4 and an anesthesiologist is responsible for their supervision, with the same physician-to-patient ratio of the CCU. The required LOS at the PACU is determined by the treating physician.

Both mechanical ventilation and non-invasive ventilation are available in the PACU, in case of emergency. However, if indicated, the patient then needs to be transferred to ICU.

In addition, the PACU anesthesiologist might be employed in other procedures, including central line positioning and loco-regional anesthesia in patients undergoing surgery.

In both settings, arterial and central venous lines that were placed intraoperatively could be used for invasive cardiovascular monitoring and support with vasopressors and/or inotropes.

### Cost analysis

The total cost includes all the costs incurred by the production unit, PACU and ICU. Some expenses are fixed (not related to activity), whereas other expenses are variable (related to activity). Shared costs are composed of direct and indirect expenses. Direct expenses are related to the production cost of the unit. We selected the following as direct expenses: wages (medical doctors, nurses and non-medical staff), medications, other single use devices, lease, maintenance and servicing of medical devices, and finally the use of an external laboratory. Indirect expenses are not directly related to the cost of production but are necessary for its realization and consist mainly of maintenance of medical equipment and pharmacy.

This cost analysis was conducted by the Finance Department of our hospital.

The expenses assessed included:

- Personnel costs: the salary of staff members was calculated as per full job time, according to the most recent collective labor agreement. For each professional involved in each unit, we identified the mean hourly wage that was then divided to the number of patients assisted in each setting, as shown in Table 5.
- Costs of single use medical devices and laboratory tests: a checklist of single use medical devices adopted for each patient admitted in the PACU and the ICU was realized, aiming at providing the overall costs of single use medical devices to patient (Table 5).

Similarly, the admission lab tests costs are displayed in Table 1, whereas the lab tests profile operated in each unit is listed in Appendix A (Table A4).

**Table 1:** Cost analysis for ICU and PACU

	ICU costs^a^			PACU costs^a^		
Personnel^b^						
	Ratio to pt	Hourly costs	Hourly costs/pt	Ratio to pt	Hourly costs	Hourly costs/pt
Physician	1:4	43.3	10.82	1:4	43.3	10.82
Nurse	1:2	20.76	10.38	1:4	20.76	5.19
CNA	1:12	15.68	1.3	1:4	15.68	3.92
Overall hourly costs to pt			22.5			19.93
Medical devices and labs						
	Mean costs			Mean costs		
Ventimask	0.88			0.88		
Intrafix® SafeSet	1.16			1.16		
Infusomat® Space Line	10.38			–		
Original Perfusor® Line	1.22			–		
ABG analysis syringe	0.38			–		
Syringe for IV infusion	0.12			–		
Respiratory humidifier and nasal cannula	1.79			1.79		
Nine disposable ECG electrodes	0.88			0.88		
CVP Manometer^c^	8.42			–		
Urine meter^c^	6.26			–		
Disposable pulse oximeter^c^	14.55			–		
Disposable expanse polyurethan pillow	5.64			–		
Disposable protective coat	0.5			–		
Dial flow IV infusion set	–			0.6		
Warming blanket	–			7.32		
Disposable emesis basin	0.08			0.08		
Clinical chart divider	0.32			–		
Admission labs	56.05			28.6		
Overall costs to pt	108.6			41.3		
Medical equipment						
	Maintenance costs/y		Amortization/y	Maintenance costs/y		Amortization/y
Mindray® multi-parameter monitor	70.76		300	70.76		300
Hourly cost to pt	370.76 ÷ (365 d * 24 h) = 0.04^d^			370.76 ÷ (253 d * 12 h) = 0.11^d^		
	Maintenance costs/y		Amortization/y			
Infusion pump^e^	84.77		294	–		
Hourly cost to pt	378.77 ÷ (365 d * 24 h) = 0.04^d^			–		
Other	Daily rental costs					
Anti-decubitus mattress^f^	3.18			–		
Overall costs to pt	134.36			61.34		

^a^All costs are expressed in Euros

^b^According to the most recent collective labor agreement

^c^These medical devices are the same as those used during the surgical procedure for each patient admitted to the PACU

^d^Calculated according to the overall days and hours of opening for each Unit during a solar year

^e^In the ICU, all intravenous therapies are administered via an infusion pump

^f^Following surgery, each ICU patient is transferred using a dedicated bed equipped with an anti-decubitus mattress, which remains in use for the entire duration of their critical care stay. In contrast, PACU patients are transferred to this unit on standard ward beds that lack this specific mattress

*ICU* intensive care unit, *PACU* post anesthesia care unit, *CNA* certified nursing assistant, *ABG* arterial blood gas, *IV* intravenous, *ECG* electrocardiogram, *CVP* central venous pressure

Since the PACU is administratively and financially integrated with the ICU, the absence of a separate budget for this unit drives a resource allocation strategy centered on cost containment, prioritizing cost-effective, disposable medical devices and the reutilization of compatible equipment from surgical procedures, a practice aligned with the typically short duration of patient stay in this unit.

- Costs of maintenance and amortization of medical equipment: in cooperation with the department of clinical engineering, we identified the annual costs of maintenance and the amortization for each medical equipment utilized in the PACU and the ICU. Then, we divided these annual costs to the time of opening/year, expressed in hours, for each unit evaluated, with the scope to obtain an hourly cost to patient, as explained in Table 5.
- Costs of pharmacy: after analyzing the therapies administered during the postoperative monitoring of each patient included in the study, we did not identify peculiar differences, in terms of pharmaceutical costs, in the setting of the PACU and the ICU. Accordingly, we decided to omit this voice from the overall cost analysis.

### Statistical analysis

Frequency distributions were determined for baseline categorical variables, and the arithmetic mean along with standard error (± SE) was calculated for baseline continuous variables [with median and corresponding interquartile (IQ) range being used for baseline continuous variables having skewed distributions].

Tests of association were performed using Pearson (uncorrected) chi-squared tests for dichotomous baseline variables and standard *t* tests or Mann–Whitney tests as appropriate for continuous baseline variables (using natural logarithmic transformed values for skewed distributions).

Multivariable analysis was performed using ordinary least squares (OLS) regressions, with statistical significance set at *p* value < 0.05.

The statistical analysis was performed using IBM SPSS Statistics for Windows, version 24 (IBM Corp., Armonk, NY, USA).

## Results

### Comparison of group ICU 1 and PACU

#### Univariable comparisons of baseline variables

No significant differences were observed in the demographic characteristics, BMI, comorbidities index according to Charlson classification and preoperative diagnosis between the two groups, whereas ASA scores were significantly less favorable in the PACU group as compared to the ICU (*p* = 0.003) (Table 2).

**Table 2:** Comparisons of baseline variables by group (ICU 1 vs PACU)^a^

	ICU 1 group (*n* = 49)	PACU group (*n* = 62)	*p* value
Age (years), median (IQ range)	71.5 (65.3–76.4)	67.7 (58.8–76.3)	0.21
Male, % (*n*)	61.2 (30)	58.1 (36)	0.74
BMI, median (IQ range)	24.8 (23.3–27.2)	24.5 (22.8–27.7)	0.60
ASA score, % (*n*)			
1	22.4 (11)	6.5 (4)	0.003
2	55.1 (27)	43.5 (27)	
3	20.4 (10)	50 (31)	
4	2.1 (1)	0 (0)	
Charlson index, median (IQ range)	6 (5–8)	7.5 (5–9)	0.12
Preoperative diagnosis, % (*n*)			
Primary benign liver neoplasm	4.1 (2)	12.9 (8)	0.12
Primary malignant liver neoplasm	38.8 (19)	46.8 (29)	
Secondary liver neoplasm	20.4 (10)	21 (13)	
Pancreatic neoplasm^b^	36.7 (18)	19.3 (12)	
Type of surgery, % (*n*)			
Hepatic resection [7]			
Major	20.4 (10)	9.7 (6)	0.007
Minor	42.9 (21)	72.6 (45)	
Pancreatic resection	36.7 (18)	17.7 (11)	
Surgical technique, % (*n*)			
Open	57.1 (28)	29 (18)	0.003
Mini-invasive	42.9 (21)	71 (44)	
Duration of monitoring (h), median (IQ range)	18 (15.8–19.7)	3 (2.3–4)	< 0.0001
Morbidity			
Clavien–Dindo grade, % (*n*)			
1	0 (0)	3.2 (2)	0.004
2	75.5 (37)	95.2 (59)	
3a	18.4 (9)	1.6 (1)	
3b	4.1 (2)	0 (0)	
4	2 (1)	0 (0)	
5	0 (0)	0 (0)	
Clavien–Dindo grade ≥ 3, % (*n*)	24.5 (12)	1.6 (1)	< 0.0001
CCI, median (IQ range)	32 (24.2–40.3)	25.7 (24.2–33.2)	0.008

^a^Mean ± SD if continuous [median (IQ range) for skewed distributions]; percentage with characteristic if categorical

^b^Including pancreatic tumors, duodenal adenocarcinoma and cholangiocarcinoma of the distal intrapancreatic bile duct

*ICU* intensive care unit, *PACU* post anesthesia care unit, *BMI* body mass index, *ASA* American Society of Anesthesiologists, *CCI* comprehensive complication index

Regarding the operative variables analyzed, significantly higher percentages of major hepatic resections and pancreatic resections performed through an open approach were reported in the ICU group (*p* = 0.007 and 0.003, respectively) (Table 2).

Similarly, median duration of monitoring was significantly longer among patients admitted to the ICU after surgery, compared to those surveilled in the PACU (*p* < 0.0001) (Table 2).

Regarding overall postoperative morbidity, the percentages of patients developing a major complication as classified by Clavien–Dindo grade ≥ 3 and consequently the median CCI were significantly major in the ICU group (*p* < 0.0001 and 0.008, respectively), as outlined in Table 2.

#### Univariable comparisons of outcome variables

In comparing postoperative outcomes by group (Table 3), significantly higher median costs of monitoring were documented among those patients whose surveillance was carried out in the ICU, compared to those monitored in the PACU (*p* < 0.0001).

**Table 3:** Comparisons of postoperative outcomes by group (ICU 1 vs PACU)^a^

	ICU 1 group (*n* = 49)	PACU group (*n* = 62)	*p* value
Monitoring cost/pt (€), median (IQ range)	503.1 (454.8–542.5)	101.2 (87.0–121.4)	< 0.0001
Length of hospital stay (days), median (IQ range)	7 (5–10)	4 (3–5.2)	< 0.0001

^a^Mean ± SD if continuous [median (IQ range) for skewed distributions]; percentage with characteristic if categorical

*ICU* intensive care unit, *PACU* post anesthesia care unit

Likewise, the LOS was significantly longer in this group (*p* < 0.0001), as shown in Table 3.

#### Subgroup analysis

A subgroup analysis was performed by evaluating several baseline variables across the following subgroups of patients: age ≥ 70 years, Charlson Comorbidity Index < 6, ASA score ≥ 3, minor hepatic resections and pancreatectomies, for both the outcomes investigated: costs of postoperative monitoring and LOS, aiming at better addressing the cost effectiveness of the PACU.

The case mix analysis confirmed the results emerged at the overall analysis, as displayed in Appendix A (Tables A5 and A6).

#### Multivariable analysis results

Multivariable analysis of the monitoring cost revealed two independent predictors of decreased costs: postoperative surveillance in the PACU (*p* < 0.0001) and shorter duration of monitoring (*p* < 0.0001), as outlined in Table 4.

**Table 4:** Multivariable analysis results

Predictors of monitoring cost/pt		
Variable	*p* value	Odds ratio (95% CI)
Postoperative surveillance in PACU	< 0.0001	−98.37 (−96.36 to −100.37)
Duration of monitoring	< 0.0001	20.236 (20.199 to 20.274)

Predictors of length of hospital stay		
Variable	*p* value	Odds Ratio (95% CI)
Clavien–Dindo ≥ 3	0.002	4.557 (1.736 to 7.377)
CCI	< 0.0001	0.210 (0.104 to 0.316)

*PACU* post anesthesia care unit, *CCI* comprehensive complication index

Finally, the occurrence of severe postoperative complications, i.e., Clavien–Dindo grade ≥ 3, and thus the CCI, represented the unique predictors of prolonged LOS (*p* = 0.002 and < 0.0001, respectively) (Table 4).

### Comparison of group ICU 2 and PACU

#### Univariable comparisons of baseline variables

No significant differences were observed in the demographic characteristics, BMI, comorbidities index according to Charlson classification and preoperative diagnosis between the two groups, whereas ASA scores were significantly less favorable in the PACU group as compared to the ICU (*p* = 0.009) (Table 5).

**Table 5:** Comparisons of baseline variables by group (ICU 2 vs PACU)^a^

	ICU 2 group (*n* = 60)	PACU group (*n* = 62)	*p* value
Age (years), median (IQ range)	68.7 (60.9–76.4)	67.7 (58.8–76.3)	0.85
Male, % (*n*)	70 (42)	58.1 (36)	0.17
BMI, median (IQ range)	26.2 (23.5–28.3)	24.5 (22.8–27.7)	0.10
ASA score, % (*n*)			
1	21.7 (13)	6.5 (4)	0.009
2	48.3 (29)	43.5 (27)	
3	26.7 (16)	50 (31)	
4	3.3 (2)	0 (0)	
Charlson index, median (IQ range)	6.5 (5–8)	7.5 (5–9)	0.31
Preoperative diagnosis, % (*n*)			
Primary benign liver neoplasm	6.7 (4)	12.9 (8)	0.28
Primary malignant liver neoplasm	41.7 (25)	46.8 (29)	
Secondary liver neoplasm	18.3 (11)	21 (13)	
Pancreatic neoplasm^b^	33.3 (20)	19.3 (12)	
Type of surgery, % (*n*)			
Hepatic resection [7]			
Major	11.7 (7)	9.7 (6)	0.06
Minor	53.3 (32)	72.6 (45)	
Pancreatic resection, % (*n*)	35 (21)	17.7 (11)	
Surgical technique, % (*n*)			
Open	58.3 (35)	29 (18)	0.001
Mini-invasive	41.7 (25)	71 (44)	
Duration of monitoring (h), median (IQ range)	17.5 (16–19.3)	3 (2.3–4)	< 0.0001
Morbidity			
Clavien–Dindo, % (*n*)			
1	0 (0)	3.2 (2)	0.24
2	91.7 (55)	95.2 (59)	
3a	3.3 (2)	1.6 (1)	
3b	1.7 (1)	0 (0)	
4	3.3 (2)	0 (0)	
5	0 (0)	0 (0)	
Clavien–Dindo grade ≥ 3, % (*n*)	8.3 (5)	1.6 (1)	0.09
CCI, median (IQ range)	25.7 (24.2–38.2)	25.7 (24.2–33.2)	0.08

^a^Mean ± SD if continuous [median (IQ range) for skewed distributions]; percentage with characteristic if categorical

^b^Including pancreatic tumors, duodenal adenocarcinoma and cholangiocarcinoma of the distal intrapancreatic bile duct

*ICU* intensive care unit, *PACU* post anesthesia care unit, *BMI* body mass index, *ASA* American Society of Anesthesiologists, *CCI* comprehensive complication index

Regarding the operative variables analyzed, significant differences were documented exclusively in the surgical technique adopted, with higher percentages of interventions carried out by a minimally invasive approach among those patients admitted to the PACU after surgery (*p* = 0.001), though a non-significant trend toward more pancreatectomies was recorded among ICU controls, as outlined in Table 5.

Likewise, median duration of monitoring was significantly longer in this group, in comparison with the PACU group (*p* < 0.0001) (Table 5).

On the other hand, when analyzing the postoperative morbidity, no significant difference was documented between the two groups, as emerged by the rate of complications according to the Clavien–Dindo grade and by the CCI (Table 5).

#### Univariable comparisons of outcome variables

In comparing postoperative outcomes by group (Table 6), significantly higher median costs of monitoring were documented among those patients whose surveillance was carried out in the ICU, compared to those monitored in the PACU (*p* < 0.0001).

**Table 6:** Comparisons of postoperative outcomes by group (ICU 2 vs PACU)^a^

	ICU 2 group (*n* = 60)	PACU group (*n* = 62)	*p* value
Monitoring cost/pz (€), median (IQ range)	491.9 (458.1–531.8)	101.2 (87.0–121.4)	< 0.0001
Length of hospital stay (days), median (IQ range)	5.5 (4–8)	4 (3–5.2)	< 0.0001

^a^Mean ± SD if continuous [median (IQ range) for skewed distributions]; percentage with characteristic if categorical

*ICU* intensive care unit, *PACU* post anesthesia care unit

Likewise, the LOS was significantly longer in this group (*p* < 0.0001), as displayed in Table 6.

#### Subgroup analysis

A subgroup analysis was performed by evaluating several baseline variables across the following subgroups of patients: age ≥ 70 years, Charlson Comorbidity Index < 6, ASA score ≥ 3, minor hepatic resections and pancreatectomies, for both the outcomes investigated: costs of postoperative monitoring and LOS, aiming at better addressing the cost effectiveness of the PACU.

The case mix analysis confirmed the results emerged at the overall analysis, as displayed in Appendix A (Tables A7 and A8).

#### Multivariable analysis results

Multivariable analysis of the monitoring cost revealed three independent predictors of decreased costs: postoperative surveillance in the PACU (*p* < 0.0001), shorter duration of monitoring (*p* < 0.0001) and male sex (*p* = 0.028), whereas the occurrence of severe postoperative complications, i.e., Clavien–Dindo grade ≥ 3, emerged as an independent predictor of increased costs of monitoring (*p* = 0.001), as outlined in Table 7.

**Table 7:** Multivariable analysis results

Predictors of monitoring cost/pt		
Variable	*p* value	Odds ratio (95% CI)
Postoperative surveillance in PACU	< 0.0001	−98.37 (−96.36 to −100.37)
Duration of monitoring	< 0.0001	20.236 (20.199 to 20.274)
Male sex	0.028	−2.038 (−0.223 to −3.852)
Clavien–Dindo grade ≥ 3	0.001	8.664 (3.576 to 13.752)

Predictors of length of hospital stay		
Variable	*p* value	Odds ratio (95% CI)
CCI	< 0.0001	0.538 (0.426 to 0.650)

*PACU* post anesthesia care unit, *CCI* comprehensive complication index

Finally, the CCI represented the unique predictor of prolonged LOS (*p* < 0.0001) (Table 7).

## Discussion

To the best of our knowledge, this is the first study assessing the costs of postoperative monitoring among patients admitted to the PACU and the ICU after elective hepatobiliary surgery, demonstrating significant benefits in terms of costs for the PACU, without negatively affecting the overall morbidity rate and LOS.

Indeed, since several advantages [1, 8, 9] and drawbacks [10–15] of the PACU have been clearly reported by different Authors, we decided to focus our attention on investigating the costs associated with postoperative monitoring in the PACU and in the ICU applied to a population of patients undergoing elective HPB surgeries at our tertiary teaching hospital. Concomitantly, we investigated the overall morbidity rate and the LOS in both groups.

First, when analyzing both groups separately, “ICU 1” and “ICU 2”, the costs of postoperative monitoring in the CCU were reported significantly higher as compared to the surveillance in the PACU. Similar conclusions were reached regarding the secondary endpoint investigated, the overall LOS.

Nevertheless, several baseline variables tested differed significantly between the PACU and the first ICU group, particularly concerning the type of surgeries carried out and the technique adopted, with more complex interventions performed in an open fashion in the ICU historical controls.

The main reason underlying these discrepancies might be identified in the absence of the PACU itself during the first period evaluated, driving anesthesiologists to address those patients at highest risk of perioperative complications because of the surgical complexity, to critical care surveillance rather than directly to the general SW.

Indeed, in line with a recent multicenter cohort study conducted by Campbell et al. [8], grades of surgical severity and magnitude together with intraoperative blood loss emerged as significant factors predicting postoperative critical care admission after inpatient surgery, with HPB and thoracic surgery identified as the only specialties carrying an increased risk of postoperative surveillance in ICUs.

Accordingly, the pre-PACU group of patients directly admitted to the CCU consisted of more complex surgeries, particularly pancreaticoduodenectomies (PD) and major hepatic resections, performed principally by an open method and thus at higher risk of perioperative morbidity.

On the other hand, the ICU 2 and the PACU groups, that refer to the same period of time, appeared considerably more homogeneous, the only differences being identified in the ASA class and surgical technique, with higher scores of anesthesiologist risk reported in the second group and once again more surgeries completed by an open fashion in the first group.

Regarding the latter point, a trend, though not significant, toward more pancreatic resections undergoing postoperative ICU monitoring, was still noted in contemporary controls that might explain the significance found in the surgical technique adopted, in line with the still ongoing tendency to perform major pancreatic surgeries, i.e., PD and total pancreatectomies, by a laparotomic approach at our institution.

Indeed, while patient-level factors remain the most influential drivers of a decision to admit a patient to critical care, in agreement with recent guidelines recommending that noncardiac surgery patients with a predicted 30-day mortality of more than 5% may benefit from ICU postoperative care [16], clinicians’ perceived requirement for critical care might exert a subtle external influence on decision-making in high-risk surgical patients, particularly when a critical care bed is available [8].

Thus, it is still common practice in many institutions to routinely admit patients to an ICU after major pancreatic surgery, exclusively for standard short-term monitoring, i.e., inferior to 24 h [17], though avoiding immediate postoperative critical care admission after robotic PD has been recently reported as a safe and cost-saving measure, without increased risk of delayed ICU transfer [18].

In addition, aiming at limiting the effect of potential biases related to the above-mentioned drawbacks, we decided to perform a case mix analysis by evaluating several baseline variables across specific subgroups of patients that confirmed the cost-effectiveness of the PACU for both the outcomes investigated.

With regard to the perioperative morbidity rate, those patients monitored in our PACU and then transferred to SW, experienced similar rates and degrees of complications, of their contemporary ICU controls, after elective HPB surgery, in agreement with Wurth et al. that reported very low incidences of relevant early complications, i.e., those occurring within 24 h, when, after a median duration of stay in the PACU of 285 min, patients were transferred to the floor [19].

In addition, a recent systematic review and metanalysis on the outcomes following EPCU admission in noncardiac surgery, reported lower mortality among patients managed in these units (2%, 95% CI 1–4%) vs those managed in ICUs (8%, 95% CI 4–14%) (*P* < 0.01), affirming the complete safety of PACUs [1].

Nevertheless, in this study, we could not conduct any mortality rate analysis, since no deaths were recorded in both groups.

In all the groups investigated, the duration of postoperative monitoring resulted significantly longer when surveillance was carried out in the ICU compared to the PACU, principally related to the inherent organization of both units at our institution. Indeed, as stated above, patients admitted to our CCU after elective surgeries, even though for a mere postoperative observation, generally spent the first night there, before being transferred to the SW on postoperative day 1, while those monitored in the PACU must be discharged to the SW by 8 p.m. of the same surgery day, due to unavailability of this unit during night-time.

Since, in the cost analysis performed by the Finance Department of our hospital, some direct expenses, and above all the personnel costs, were quantified as hourly costs, an unavoidable direct relationship between duration of monitoring and associated costs exists, representing thus a potential bias in this study.

Accordingly, aiming at limiting this effect and at focusing on the short-term postoperative recovery care, we excluded all those patients with ICU stay longer than 24 h, considering it a nonroutine critical care monitoring, due to the development of early complications.

Regarding the secondary outcome analyzed, when comparing both historical and contemporary ICU controls to the PACU group, it emerged a significant reduction in the LOS among PACU admitted patients, in agreement with several studies [2, 17, 18].

On the other hand, the previously cited review and systemic metanalysis on the outcomes following EPCU admission in noncardiac surgery, showed similar overall LOS after transferring the patient to an EPCU or ICU (8.6 days, 95% CI 5.9–11.3 days) [1], whereas in the propensity-matched cohort study conducted by Thevathasan et al. [20], admission to a CCU was associated with decreased hospital LOS and costs only in case of high propensity for postoperative intensive care needs.

However, since considerable heterogeneity in operational definitions of the different units of postoperative care evaluated, types of surgeries included, specific comorbidities and admission criteria were reported among the studies cited, those findings necessitate cautious interpretation.

Indeed, in this study, this association between PACU’s postoperative admission and overall hospital LOS, was no longer validated at multivariable analysis, being the only significant predictor of this outcome, the occurrence of postoperative complications.

On the other hand, the surveillance in PACU after surgery was confirmed as the most important factor in reducing the costs associated with postoperative monitoring, though the overall duration of monitoring was observed as the other highly significant predictor of costs in this study.

However, this finding should not lead one to conclude that reducing the duration of monitoring might actually decrease the costs associated with monitoring. In fact, a longer duration of postoperative surveillance was documented in both historical and contemporary ICU controls compared to the PACU admitted patients, strongly supporting the different management of both units at our hospital, as described in other studies [17]. Thus, the value of longer duration of monitoring in terms of implying greater costs appears to be more a reflection of the inherent organization of the PACU and ICU at our hospital-level rather than the definitive concurrence in overall costs.

Finally, at multivariable analysis, when considering exclusively the group of contemporary ICU controls, male sex was associated with cost savings in terms of postoperative monitoring, differently from what stated by Evans et al. that attributed to male patients major critical care charges due to a greater burden of cardiovascular diseases [21], which represent the largest proportion of these, whereas occurrence of severe postoperative complications, according to Clavien–Dindo grades ≥ 3, represented another significant risk factor for increased monitoring costs.

There are several limitations to this study, particularly that of cause and effect. As previously described, patients admitted to the ICU after elective HPB surgery tend to present a longer stay compared to those surveilled in the PACU, before being transferred to the SW, serving as a potential bias for increased monitoring costs. However, it appears how a longer duration of monitoring by itself might not be actually considered a risk factor for increased costs, but more appropriately a result of a different management of those patients. Accordingly, we tried to mitigate this effect by including only those patients with a stay in the CCU < 24 h.

Second, although the data were prospectively collected and stored in our hospital database, the analysis performed was a single‐center retrospective cohort study. Nevertheless, this study was focused on a single surgical specialty, in contrast to other studies grouping several different types of surgeries.

In addition, single‐center studies also allow for the collection and investigation of a larger number of potentially relevant baseline variables, which may contribute to a more accurate assessment of the associations between those variables and prespecified study outcomes.

## Conclusions

The PACU represented a safe alternative to the ICU for postoperative monitoring in patients undergoing elective HPB surgeries. Postoperative surveillance in this specific unit resulted the strongest multivariable predictor of lower monitoring costs, followed by shorter duration of monitoring, male gender and uneventful postoperative course.

Further research is needed to predict more clearly, and with better objectifiable criteria, whether a patient will benefit from postoperative admission to an ICU or whether monitoring for a few hours in the PACU might be sufficient.

## Supplementary Information

Below is the link to the electronic supplementary material.

Supplementary file1 (DOCX 36 KB)

## Data Availability

The data sets generated during and/or analyzed during the current study are available from the corresponding author on reasonable request.
