# Supplementary material for: Over-night monitoring in intensive care unit and short-term monitoring in post anesthesia care unit costs analysis after elective hepato-pancreatic–biliary surgery: a retrospective study
Source: Updates Surg. 2025 Dec 2;78(2):705–16. doi: 10.1007/s13304-025-02405-9 (PMC13212388; doi:10.1007/s13304-025-02405-9)
Supplement: Supplementary file 1 — Supplementary file1 (DOCX 36 KB) [file 13304_2025_2405_MOESM1_ESM.docx]

**Table A1. ICD-9-CM codes used for selection of patients**

| **ICD-9-CM** | **Surgical procedure** |
| --- | --- |
| **50.22** | Partial hepatectomy |
| **50.3** | Lobectomy of the liver |
| **52.6** | Total pancreatectomy |
| **52.7** | Radical pancreaticoduodenectomy |
| **52.52** | Distal pancreatectomy |
| **52.22** | Other excision or destruction of lesion or tissue of pancreas or pancreatic duct |

**Table A2. Variables analyzed**

| **Preoperative** | **Operative** | **Postoperative** |
| --- | --- | --- |
| Demographics (age and sex)  BMI  ASA score  Charlson comorbidity index  Diagnosis | Type of surgery:  Hepatic resection: major vs minor^a^  Pancreatic resection  Surgical technique:  open vs mini-invasive | Duration of monitoring in PACU and ICU  Morbidity rate  Severity of complications:  Clavien-Dindo grade and CCI  Monitoring Cost to pt  Length of hospital stay |

^a^ major: resection of ≥ 3 liver segments, minor: resection of < 3 segments ^7^

BMI, Body Mass Index; ASA, American Society of Anesthesiologists; PACU, Post Anesthesia Care Unit; ICU, Intensive Care Unit; CCI, Comprehensive Complication Index

**Table A3. ICU and PACU characteristics**

|  | **ICU** | **PACU** |
| --- | --- | --- |
| Nurse-to-patient ratio | 1:2 | 1:4 |
| Physician-to-patient ratio | 1:4 | 1:4 |
| Beds available | 12 | 4 |
| Opening hours | 7 days per week,  24 h per day | Monday to Friday,  8 a.m. to 8 p.m. |
| Admission | Direct postoperatively | Direct postoperatively |
| Medical supervision | Critical care physician | Anesthesiologist |
| Vasopressor support | Yes | Yes |
| Duration of stay | Overnight | Individualized, based on the  estimation of anesthesiologist |

ICU, Intensive Care Unit; PACU, Post-Anesthesia Care Unit

**Table A4.** **Admission lab tests and the relative costs for ICU and PACU**

|  | **ICU** | **PACU** |
| --- | --- | --- |
|  | **Costs^a^** | **Costs^a^** |
| Complete blood count | 3.15 | 3.15 |
| Serum creatinine | 1.1 | 1.1 |
| BUN | 1.1 | 1.1 |
| Serum PT | 2.3 | 2.3 |
| Serum PTT | 2.5 | 2.5 |
| Serum fibrinogen | 1.75 | 1.75 |
| Blood glucose | 1 | 1 |
| Serum bilirubin: total and direct | 2.2 | 2.2 |
| Serum ALT | 1 | 1 |
| Serum AST | 1.05 | 1.05 |
| Serum ALP | 1.05 | 1.05 |
| Serum GGT | 1.15 | 1.15 |
| Serum LDH | 1.1 | 1.1 |
| Serum CPK | 1.75 | - |
| Serum amylase | 1.25 | 1.25 |
| Serum lipase | 2.6 | 2.6 |
| Serum sodium | 0.85 | 0.85 |
| Serum potassium | 1.25 | 1.25 |
| Serum calcium | 1.1 | 1.1 |
| Serum phosphate | 1 | - |
| Serum magnesium | 1.65 | - |
| Serum chloride | 1.1 | - |
| Serum albumin | 1.1 | 1.1 |
| KPC rectal swab | 21.95 | - |
| **Total** | 56.05 | 28.6 |

^a^ expressed in Euros

ICU, Intensive Care Unit; PACU, Post-Anesthesia Care Unit; BUN, Blood Urea Nitrogen; PT, Prothrombin Time; PTT, Partial Thromboplastin Time; ALT, Alanine Transaminase; AST: Aspartate Transferase; ALP, Alkaline Phosphatase; GGT, Gamma-Glutamil Transferase; LDH, Lactate Dehydrogenase; CPK, Creatine phosphokinase; KPC, Klebsiella Pneumoniae Carbapenemase

**Table A5.** **Subgroup analysis for the outcome monitoring cost/pt**

|  | **ICU 1 group**  **(n = 49)** | **PACU group**  **(n = 62)** | ***p*-Value** |
| --- | --- | --- | --- |
| **Variable** | **Monitoring cost/pt (€), median (IQ range)** | |  |
| Age ≥ 70 (years) | 528.1 (477.8-561.9) | 103.1 (86.9-123.5) | **<0.0001** |
| Charlson index <6 | 511.1 (460.3-539.3) | 97 (88.9-113.3) | **<0.0001** |
| ASA score ≥ 3 | 528.1 (507.2-570.4) | 107.2 (89.4-123.5) | **<0.0001** |
| Minor hepatic resections | 528.1 (457.5-561.9) | 103.1 (88.4-123.5) | **<0.0001** |
| Pancreatectomies | 516.8 (477.8-537.7) | 97 (88.9-113.3) | **<0.0001** |

ICU, Intensive Care Unit; PACU, Post Anesthesia Care Unit; ASA, American Society of Anesthesiologists

**Table A6.** **Subgroup analysis for the outcome length of hospital stay**

|  | **ICU 1 group**  **(n = 49)** | **PACU group**  **(n = 62)** | ***p*-Value** |
| --- | --- | --- | --- |
| **Variable** | **Lenght of hospital stay (days), median (IQ range)** | |  |
| Age ≥ 70 (years) | 7 (5.2-14.7) | 3 (3-4.2) | **<0.0001** |
| Charlson index <6 | 8 (6-11) | 4 (3-7) | **0.001** |
| ASA score ≥ 3 | 6 (4.5-9.7) | 4 (3-6) | **0.006** |
| Minor hepatic resections | 5 (4-6.5) | 3 (3-4) | **0.0001** |
| Pancreatectomies | 8 (7-12.5) | 7 (5.2-7.7) | **0.04** |

ICU, Intensive Care Unit; PACU, Post Anesthesia Care Unit; ASA, American Society of Anesthesiologists

**Table A7.** **Subgroup analysis for the outcome monitoring cost/pt**

|  | **ICU 2 group**  **(n = 60)** | **PACU group**  **(n = 62)** | ***p*-Value** |
| --- | --- | --- | --- |
| **Variable** | **Monitoring cost/pt (€), median (IQ range)** | |  |
| Age ≥ 70 (years) | 494.2 (460.3-543.9) | 103.1 (86.9-123.5) | **<0.0001** |
| Charlson index <6 | 505.5 (469.9-548.9) | 97 (88.9-113.3) | **<0.0001** |
| ASA score ≥ 3 | 505.5 (449-539.4) | 107.2 (89.4-123.5) | **<0.0001** |
| Minor hepatic resections | 511.1 (471.6-545) | 103.1 (88.4-123.5) | **<0.0001** |
| Pancreatectomies | 505.5 (475-548.4) | 97 (88.9-113.3) | **<0.0001** |

ICU, Intensive Care Unit; PACU, Post Anesthesia Care Unit; ASA, American Society of Anesthesiologists

**Table A8.** **Subgroup analysis for the outcome length of hospital stay**

|  | **ICU 2 group**  **(n = 60)** | **PACU group**  **(n = 62)** | ***p*-Value** |
| --- | --- | --- | --- |
| **Variable** | **Lenght of hospital stay (days), median (IQ range)** | |  |
| Age ≥ 70 (years) | 6.5 (5-8) | 3 (3-4.2) | **0.0001** |
| Charlson index <6 | 7 (4-8.5) | 4 (3-7) | **0.02** |
| ASA score ≥ 3 | 7 (5-9) | 4 (3-6) | **0.002** |
| Minor hepatic resections | 5 (4-5) | 3 (3-4) | **<0.0001** |
| Pancreatectomies | 8 (7-11.7) | 7 (5.2-7.7) | **0.02** |

ICU, Intensive Care Unit; PACU, Post Anesthesia Care Unit; ASA, American Society of Anesthesiologists
